# A new genus and species of Macrosiphini (Hemiptera, Aphididae) from China, living on *Isodon eriocalyx*

**DOI:** 10.3897/zookeys.359.6219

**Published:** 2013-12-05

**Authors:** Bin Zhang, Ya-Meng Lou, Ge-Xia Qiao

**Affiliations:** 1 Key Laboratory of Zoological Systematics and Evolution, Institute of Zoology, Chinese Academy of Sciences, No. 1-5 Beichen West Road, Chaoyang District, Beijing 100101, P.R. China; 2 University of Chinese Academy of Sciences, No. 19, Yuquan Road, Shijingshan District, Beijing 100049, P.R. China; 3 Zhongguancun High School of Beijing, No.1-14 Kexueyuan South Road, Haidian District, Beijing 100080, P.R. China

**Keywords:** *Nigritergaphis*, Aphididae, new genus, new species, China, taxonomy

## Abstract

The aphid genus *Nigritergaphis* **gen. n.** is described, and *N. crassisetosa* **sp. n.** on *Isodon eriocalyx* (Dunn) Kudô (Lamiaceae) from Yunnan, China is described and illustrated. The new species curls and distorts the leaves of this important traditional Chinese medicinal plant, and is evidently specific to this host. Holotype and paratypes are deposited in the National Zoological Museum of China, Institute of Zoology, Chinese Academy of Sciences, Beijing, China.

## Introduction

*Isodon eriocalyx* (Dunn) Kudô (Lamiaceae) is an important traditional Chinese medicine plant, and has been used as a folk medicine to treat inflammation as well as reducing blood pressure. Over the past twenty years, plants in the genus *Isodon* have received considerable phytochemical and biological attention. These studies have revealed that a number of diterpenoids from genus *Isodon* possess various bioactivities, such as anti-tumor and anti-bacterial activities (Sun et al. 2006; Wang et al. 2013). *Isodon eriocalyx* is distributed mainly in southwest China, e.g. Yunnan, Sichuan, Guizhou and Guangxi provinces. According to the literature, no aphids have previously been recorded from this plant.

About a few years ago, researchers in the field of phytochemistry at the Kunming Institute of Botany found that this plant was being harmed by a small insect, causing the leaves to become curled, swollen and blistered. In 2011 some specimens were submitted for identification, and found to belong to a new genus and species of the aphid tribe Macrosiphini.

## Material and methods

All specimens examined in this study were collected at Kunming Institute of Botany, Chinese Academy of Sciences (Yunnan Province: Kunming City, Heilongtan Town) by J.X. Pu. The host plant was identified by the botanist, Prof. H. Peng of that Institute.

Aphid terminology in this paper generally follows that of Stekolshchikov and Qiao (2008). The unit of measurement is millimetres (mm).

## Results and discussion

### Nigritergaphis gen. n.

http://zoobank.org/A6C6E2DA-EF71-4F53-BCC6-E8739048FF4D

http://species-id.net/wiki/Nigritergaphis

#### Type-species.

*Nigritergaphis crassisetosa* sp. n.

#### Etymology.

The generic name, *Nigritergaphis* is feminine in gender and derived from the Latin words terms “*nigr-*” (=shining black), and “*terg-*” (=dorsum or back) combined with “*aphis*” (=plant louse).

#### Generic diagnosis.

*In apterae*: Body elliptical, of medium size. Median frontal tubercle poorly developed, antennal tubercles developed, diverging, slightly higher than median frontal tubercle, so that frons is shallowly “W”-shaped. Tergum smooth and sclerotic. Dorsal setae of body numerous, long, thick, stiff, and arising from tuberculate bases; ventral setae very sparse. Ultimate rostral segment longer than second hind tarsal segment, with 2–3 pairs of accessory setae. Eyes with relatively few facets. Antennae 5- or 6-segmented, much shorter than body, without secondary rhinaria; processus terminalis about 2–3 times longer than base of the last segment. Mesosternal furca with a short stem or separate arms. First tarsal chaetotaxy 2, 2, 2. Spiracles small, oval or reniform, open; spiracular plates oval. Marginal tubercles absent. Siphunculi short and tapering, slightly swollen towards base, with distinct imbrication and a well-developed flange, and sometimes with one seta. Cauda helmet-shaped, but slightly acute at apex, with 4 setae. Genital plate with 2 anterior setae and 10–12 posterior setae. *In alatae*: Abdominal tergites each with one pair of marginal patches and an imperfect spino-pleural dark band. Antennae 6-segmented, segments III–V with large and round secondary rhinaria. Fore wing median vein with two-forks, hind wing with two obliques, all veins without fuscous borders.

#### Taxonomic notes.

*Nigritergaphis* belongs to the tribe Macrosiphini of the family Aphididae, but has a unique combination of features justifying the erection of a new genus. Compared with other aphid genera associated with Lamiaceae, it differs by having numerous, thick long and stiff dorsal setae arising from tuberculate bases, and first tarsal chaetotaxy: 2, 2, 2. It is similar to *Roepkea* Hille Ris Lambers in the shape of the cauda, dorsal ornamentation and lack of marginal tubercles, but differs from that genus as follows: siphunculi only with weak imbrications (in *Roepkea*: ornamented with transverse rows of small spicules); body dorsum completely sclerotic and smooth (in *Roepkea*: usually dark, sclerotic, but with pale patches on the marginal areas, and with numerous small spicules). The new genus resembles *Brachycaudus* van der Goot in the shape of cauda, and in dorsal sclerotization, but may be distinguished from that genus by the following: lack of marginal abdominal tubercles (in *Brachycaudus* these are frequently present on several body segments); spiracular pores oval or reniform (in *Brachycaudus* they are circular and large); siphunculi imbricated and without a sharply limited subapical constriction (in *Brachycaudus* rather smooth, and with a sharply limited constriction below the flange). The new genus resembles *Dysaphis* Börner in the shape of spiracular pores and the shape of the cauda, but differs from *Dysaphis* as follows: abdomen of apterae with a complete sclerotic shield (in *Dysaphis* dorsal sclerotization never forms a complete shield); without spinal and marginal tubercles (in *Dysaphis* spinal and marginal tubercles are characteristically present).

### Nigritergaphis crassisetosa sp. n.

http://zoobank.org/139676D8-D790-437C-9B80-79AC01C78E02

http://species-id.net/wiki/Nigritergaphis_crassisetosa

Figures 1 –38

#### Locus typicus.

China (Yunnan: Kunming City, Heilongtan Town, Alt. 1922m, 102.44°E, 25.8°N).

#### Etymology.

The specific name, *crassisetosa* is composed of the Latin words “*crassis*” (= thick) and “*setosa*” (= covered with hairs), due to the long and thick setae of body.

#### Description.

*Apterous viviparous female*: Body elliptical, 1.42–1.58 mm long, 0.77–0.96 mm wide. Adult body black in life, nymphs dark green (Fig. 38).

#### Mounted specimens.

Body. Dorsum brown. Antennal segments I-II dark brown, segment V and base of segment VI brown, others pale. Siphunculi dark brown. For morphometric data see Table 1.

**Table 1.** Biometric data for *Nigritergaphis crassisetosa* sp. n. (measurements in mm).

	Apterous viviparous females n = 25		Alate viviparous females n=12
	Ant. 5-segmented (n=10 )	Ant. 6-segmented (n=15)	
Body length	1.42–1.58	1.43–1.56	1.50–1.64
Body width	0.82–0.96	0.77–0.89	0.69–0.79
Antenna length	0.52–0.62	0.58–0.65	0.72–1.14
Antennal segment I	0.054–0.065	0.05–0.06	0.05–0.06
Antennal segment II	0.040–0.045	0.04–0.05	0.04–0.05
Antennal segment III	0.16–0.21	0.11–0.16	0.23–0.31
Antennal segment IV	0.075–0.085	0.07–0.09	0.13–0.17
Antennal segment V	0.065–0.080	0.07–0.09	0.11–0.14
Antennal segment VIb		0.06–0.08	0.08–0.10
Processus terminalis	0.11–0.15	0.15–0.19	0.21–0.31
Length of setae on ant. seg. III	0.01–0.015	0.011–0.014	0.013–0.015
Ant. seg, III basal diameter	0.02	0.016–0.020	0.02–0.03
Ultimate rostral segment (URS)	0.09–0.095	0.08–0.09	0.08–0.09
Basal width of URS	0.045–0.055	0.04–0.05	0.04–0.05
Hind femur length	0.26–0.30	0.26–0.30	0.35–0.45
Hind tibia length	0.38–0.43	0.45–0.50	0.68–0.81
Midlength width of hind tibia	0.025–0.30	0.04–0.05	0.02–0.03
Hind tarsus segment II	0.06–0.065	0.06–0.07	0.06–0.07
Length of setae on hind tibia	0.02–0.025	0.021–0.026	0.025–0.028
Siphunculus length	0.10–0.11	0.09–0.10	0.09–0.10
Siphunculus basal width	0.045–0.07	0.05–0.07	0.04–0.05
Siphunculus distal width	0.04–0.045	0.03–0.04	0.03–0.04
Cauda length	0.075–0.10	0.07–0.08	0.08–0.09
Basal width of cauda	0.075–0.09	0.09–0.11	0.09–0.11
Length of frontal setae	0.040–0.055	0.041–0.046	0.019–0.024
Length of marginal setae on tergum I	0.055–0.065	0.056–0.066	0.023–0.027
Length of dorsal setae on tergum VIII	0.060–0.080	0.058–0.074	0.031–0.039

**Figures 1–11.**
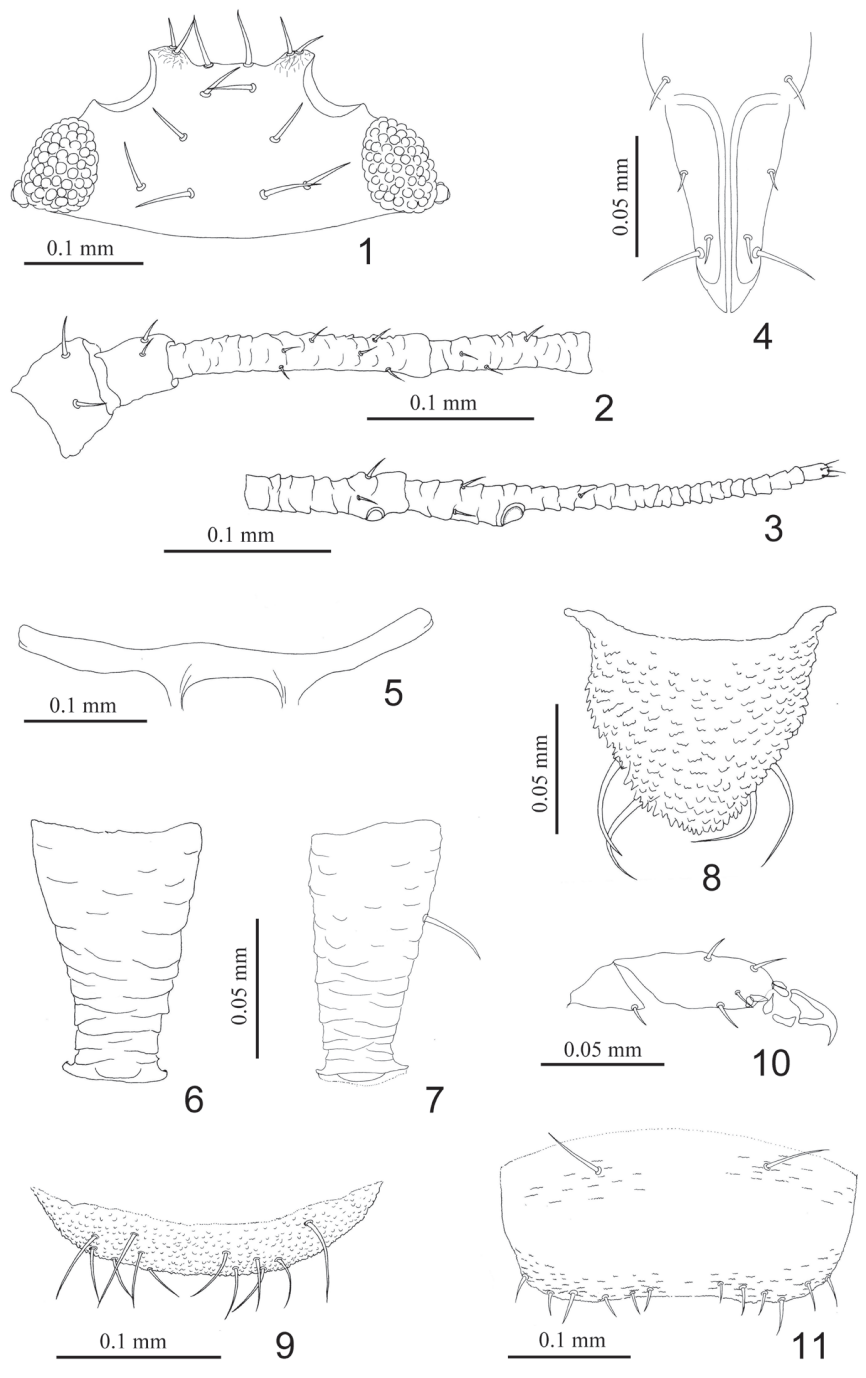
*Nigritergaphis crassisetosa* sp. n. Apterous viviparous female: **1** dorsal view of head **2** antennal segments I–IV **3** antennal segments V–VI **4** ultimate rostral segment **5** mesosternal furca **6, 7** siphunculi **8** cauda **9** anal plate **10** hind tarsal segment **11** genital plate.

Head. Smooth dorsally, except top surface of median frontal tubercle which is rough. Median frontal tubercle poorly developed, lower than antennal tubercles which are more developed and have divergent inner faces, so that frons is shallowly “W”-shaped (Figs 1, 18, 19); dorsum of head pigmented (Fig. 18), ventral surface with sparse spinules. Dorsal cephalic setae thick and stiff, arising from tuberculate bases. Head with 3 pairs of frontal setae, 1 pair of dorsal setae between antennae, and 3 pairs of dorsal setae between eyes (Figs 1, 19), frontal setae 2.0–2.8 times as long as basal diameter of antennal segment III. Eyes with about 25 facets. Antennae 5- or 6-segmented (Figs 2, 3, 20), about 0.4 of body length; segments I–II segments slightly rough, segments III–V with weak imbrications, segment VI with distinctly transverse imbrications. Processus terminalis 2.2-2.9 × base of segment VI (6-segmented antennae). Antennal setae short and pointed, segments I–VI (I-V) respectively with 4–5, 3–4, 5–7, 3–4, 3–4, 2–3+2 (3-4, 3-4, 1-3, 3-4, 1-3+3-4) setae; apex of processus terminalis with 3 or 4 setae. Setae on segment III 0.5-0.9 times as long as basal diameter of the segment. Primary rhinaria ciliated, secondary rhinaria absent (Figs 3, 20). Rostrum (Fig. 18) reaching mid-coxae; ultimate rostral segment wedge-shaped, 1.6–2.0 times as long as its basal width, 1.3–1.6 times as long as second hind tarsal segment, with 2-3 accessory setae.

Thorax. Dorsum of thorax imperfectly pigmented (Fig. 18). Pronotum with 3 pairs of spinal, 1 pair of pleural and 2 pairs of marginal setae. Mesosternal furca (Figs 5, 22) with a short stem or separate arms. Base of femora with 2-3 small round pseudo-sensoria. Hind femur 1.9–2.3 times longer than antennal segment III (when antennae 6-segmented). Hind tibia 0.28–0.34 times as long as body. Setae on legs long and pointed, setae on hind tibiae 0.7–0.9 times as long as middle diameter of the segment. First tarsal chaetotaxy: 2, 2, 2.

Abdomen. Abdominal tergites smooth, and sclerotic (Fig. 18). Dorsal setae of body numerous, long, thick and stiff, arising from tuberculate base. Marginal setae slightly longer than spinal and pleural setae. Ventral setae very sparse, fine and pointed, distinctly shorter than dorsal setae. Abdominal tergite I with 24–38 dorsal setae, tergite VIII with 4 setae, occasionally 3. Length of marginal setae on tergite I 3.0–4.1 times as long as basal diameter of antennal segment III; dorsal setae on tergite VIII 3.0–4.5 times as long as basal diameter of antennal segment III. Spiracles small, oval or reniform, opened; spiracular plates oval. Siphunculi (Figs 6, 7, 23, 24) short and tapering, with distinct imbrication and a well-developed flange, 1.5–2.5 times as long as their basal width, 1.1–1.5 times as long as cauda; siphunculi of 7 specimens with 1 long seta (Figs 7, 24). Cauda (Figs 8, 25) nearly helmet-shaped, with short rows of spinules; 0.8–1.2 times as long as basal width, with 4 long curved setae. Anal plate semi-circular, with short rows of large spinules; with 10–13 setae. Genital plate (Figs 11, 28) transversely oval, with transverse rows of spinules; 2 anterior setae, 10–12 posterior setae along margin uniformly distributed.

*Alate viviparous female*: Body elliptical, 1.50–1.64 mm long, 0.69–0.79 mm wide. For morphometric data see Table 1.

#### Mounted specimens.

**Figures 12–17.**
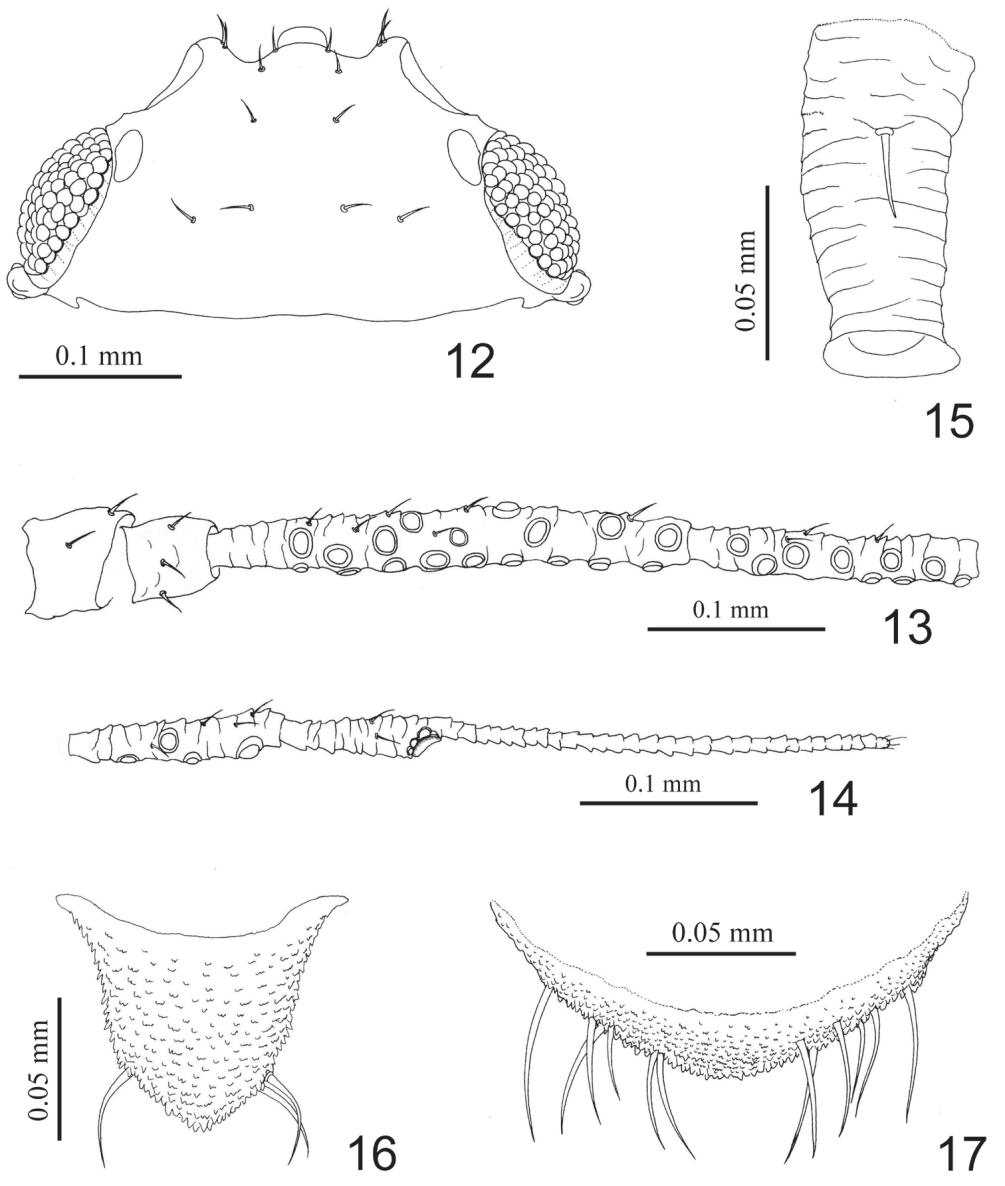
*Nigritergaphis crassisetosa* sp. n. Alate viviparous female: **12** dorsal view of head **13** antennal segments I–IV **14** antennal segments V–VI **15** siphunculus **16** cauda **17** anal plate.

Head. Median frontal tubercle weakly developed, slightly lower than antennal tubercles which are more developed with divergent inner faces, frons “W”-shaped (Figs 12, 30); dorsum of head pigmented (Figs 29, 30); ventral surface of head with sparse spinules. Head with 3 pairs of frontal setae, 2 pairs of dorsal setae between antennae, and 2 pairs of setae between eyes (Figs 12, 30). Frontal setae 0.9-1.0 times as long as basal diameter of antennal segment III. Antennae 6-segmented (Figs 13, 14, 31) 0.5–0.7 times as long as body; ventral side of segment I with weak spinulose imbrications, ventral sides of segment II-VI with imbrications. Antennal setae pointed, segments I–VI each with 4-5, 4-5, 6-11, 3-5, 3-5, 2+0, respectively, apex of processus terminalis with 3-4 setae. Setae on segment III 0.5–0.7 times as long as basal diameter of the segment. Primary rhinaria ciliated. Secondary rhinaria large and round, segment III with 18–34, segment VI with 7–12, and segment V with 1–3 (Figs 13, 14, 31). Ultimate rostral segment 1.8–2.2 times as long as its basal width, 1.3–1.4 times as long as second hind tarsal segment, with 2-4 pairs of accessory setae.

Thorax. Dorsum of thorax completely sclerotized (Fig. 29). Pronotum with 2–3 spinal setae, 1 pair of pleural and 1 pair of marginal setae. Base of femora with 3–5 small and round pseudo-sensoria, basal 1/3 rather thin; hind femur 1.4–1.7 times as long as antennal segment III. Hind tibia 0.4–0.5 times as long as body. Setae on hind tibiae 0.9–1.0 times as long as middle diameter of the segment.

Abdomen. Abdominal tergites each with one pair of marginal patches and an imperfect dark spino-pleural band; those on tergites I-III and VIII narrow; marginal patches with spinulose short stripes (Fig. 29). Dorsal setae numerous, thick, stiff and pointed, arising from tuberculate base; ventral setae very sparse, fine and pointed, approx. as long as dorsal setae. Abdominal tergite I with 12–16 spino-pleural and 4–6 marginal setae, tergite II with 14–20 spino-pleural and 10–14 marginal setae, tergite III with 14–22 spino-pleural and 11–14 marginal setae, tergite IV with 14–21 spino-pleural and 11–14 marginal setae, tergite V with 14–17 spino-pleural and 8–10 marginal setae, tergite VI with 8–13 spino-pleural and 8–10 marginal setae, tergite VII with 7–10 dorsal setae, tergite VIII with 3–5 setae. Marginal setae on tergite I 0.9–1.3 times as long as basal diameter of antennal segment III; dorsal setae on tergite VIII 1.3–1.9 times as long as basal diameter of antennal segment III. Siphunculi similar in shape to those of apterous viviparous females; 5 of 12 specimens each with 1 long seta on siphunculi (Figs 15, 32); 2.3–2.6 times as long as their basal width, 1.0–1.2 times as long as cauda.

#### Type series.

Holotype: apterous viviparous female, China: Yunnan Province, Kunming City (Heilongtan Town), 11 Dec. 2012, No. Y9259-1-2-2, on *Isodon eriocalyx*, coll. J.X. Pu. Paratypes: 12 apterous viviparous females and 11 alate viviparous females, with the same collection data as holotype; 12 apterous viviparous females and1 alate viviparous female, 27 Oct. 2011, No. Y9162, on *Isodon eriocalyx*, coll. J.X. Pu. The holotype and paratypes of the new species are deposited in the National Zoological Museum of China, Institute of Zoology, Chinese Academy of Sciences, Beijing.

#### Biology.

The species colonizes the underside of the leaves of the host plant, *Isodon eriocalyx*, inducing the leaves to become curled, swollen or blistered to form “pseudogalls”, and causing stunting of growth (Figs 36, 37, 38).

Apterae with 5-segmented antennae and 6-segmented antennae occur at different times of year, the former in October, the latter in December. Seemingly, there is a seasonal effect on morphology in this species. No sexual morphs were observed.

**Figures 18–28.**
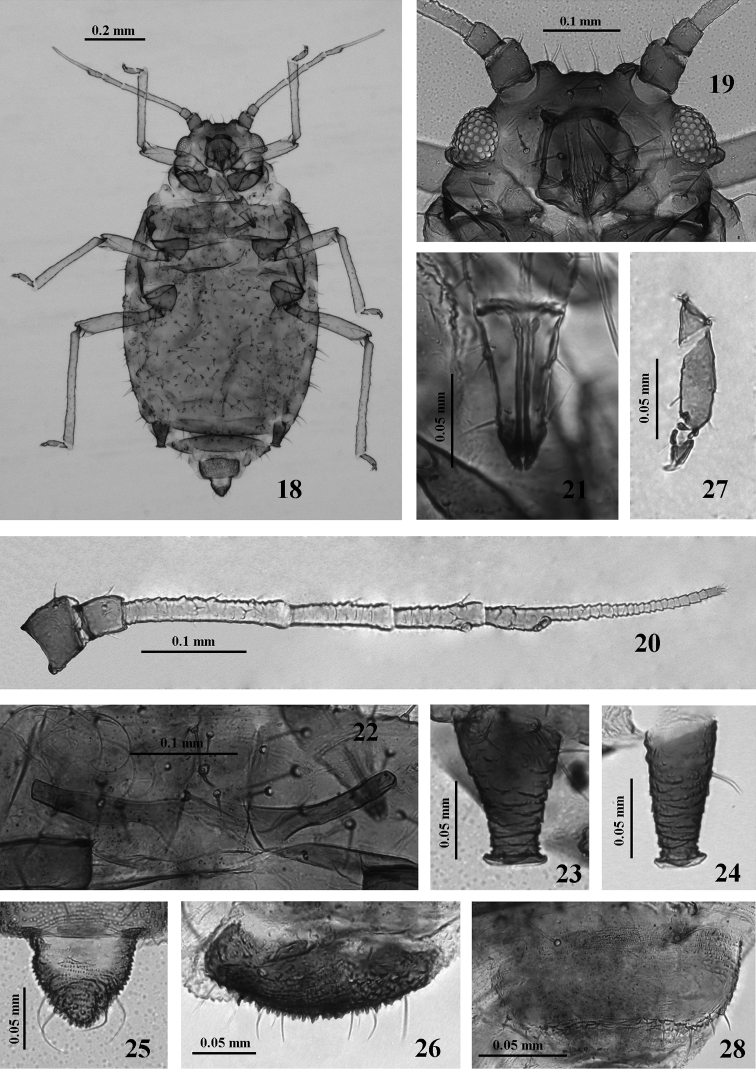
*Nigritergaphis crassisetosa* sp. n. Apterous viviparous female: **18** dorsal view of body **19** dorsal view of head **20** antennal segments I–VI **21** ultimate rostral segment **22** mesosternal furca **23, 24** siphunculi **25** cauda **26** anal plate **27** hind tarsal segment **28** genital plate.

**Figures 29–35.**
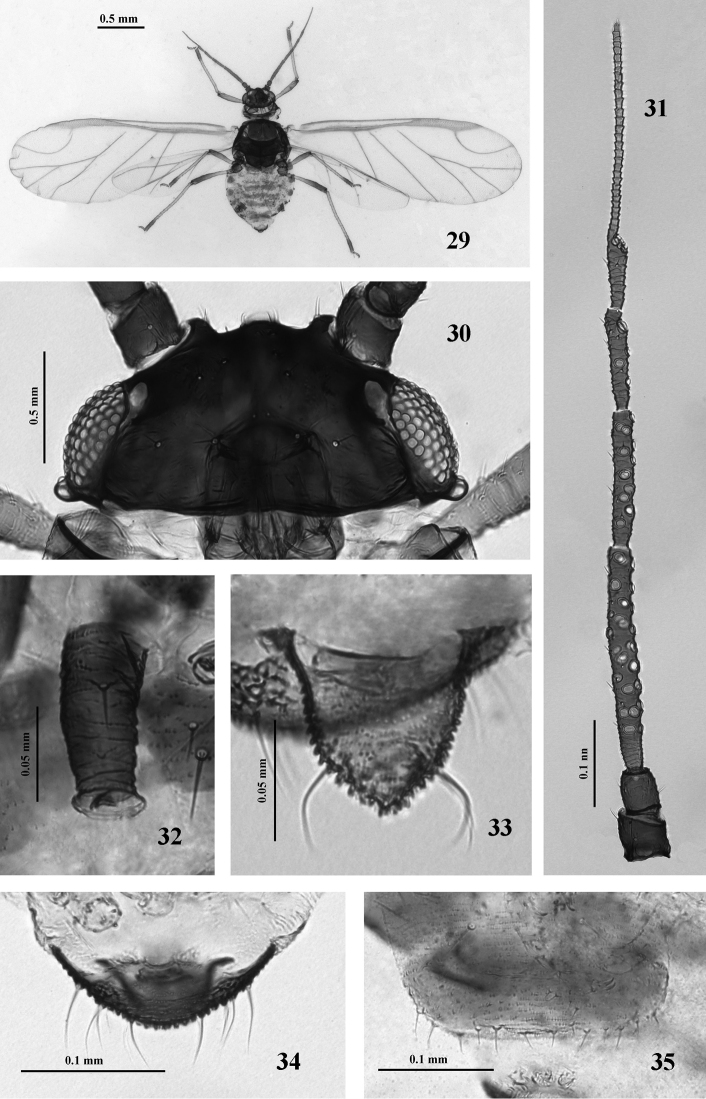
*Nigritergaphis crassisetosa* sp. n. Alate viviparous female: **29** dorsal view of body **30** dorsal view of head **31** antennal segments I–VI **32** siphunculus **33** cauda **34** anal plate **35** genital plate and gonapophyses.

**Figures 36–38.**
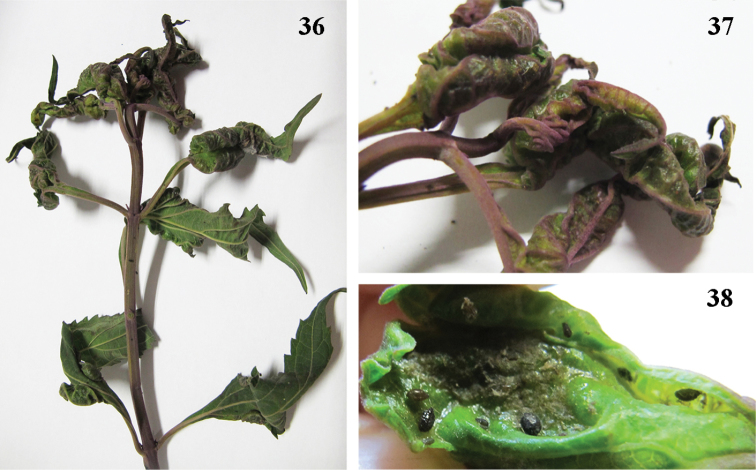
*Nigritergaphis crassisetosa* sp. n. **36** damaged shoots of host plants **37** pseudo-galls (leaf roll-like) **38** aphids in the pseudo-gall.

#### Taxonomic notes.

Blackman and Eastop (2006, 2013) provided a key to all apterous viviparous females on the plant genus *Isodon*. The new species can be inserted as an additional couplet (couplet 3a) in their key:

**Table:** 

1	SIPH strongly swollen, with maximum diameter of swollen part more than 2 × minimum diameter of stem, smooth except for a small subapical polygonal reticulation. SIPH 5.4–8.2 × cauda	*Eucarazzia elegans*
–	SIPH not swollen or much less swollen, and less than 3.5 × cauda	2
2	Dorsal hairs long, 2–4 × longer than Ant. III BD	3
–	Dorsal hairs all or mostly less than 1.5 × Ant. III BD	6
3	Cuticle of head spiculose or granulose dorsally. Dorsal hairs with blunt or pointed apices	*Eumyzus clinopodii*
–	Cuticle of head smooth. Dorsal hairs with expanded apices or pointed apices	3a
3a	Dorsal hairs with pointed apices. Ant. III without secondary rhinaria. First tarsal chaetotaxy: 2, 2, 2. SIPH 1.0–1.2 × cauda which is approximately helmet-shaped	*Nigritergaphis crassisetosa* sp. n.
–	Dorsal hairs with expanded apices. Ant. III with secondary rhinaria. First tarsal chaetotaxy: 3, 3, 3. SIPH 1.2-c.2.5 × cauda which is slender, triangular	4

Couplets 4 to 20 without modification.

## Supplementary Material

XML Treatment for Nigritergaphis

XML Treatment for Nigritergaphis crassisetosa
